# Myoglobin Cast Nephropathy Diagnosed on Renal Biopsy in a Patient Treated for Malarial Infection

**DOI:** 10.1155/2024/6764335

**Published:** 2024-02-12

**Authors:** Ramya Varadarajan, Ashmi Patel, Haneen Salah, Neil Sutaria, Roberto Barrios, Luan Truong, Lillian Gaber, Ziad M. El-Zaatari

**Affiliations:** ^1^Texas A&M Health Science Center, College Station, TX, USA; ^2^Houston Methodist, Houston, TX, USA

## Abstract

Myoglobin cast nephropathy occurs in cases of acute renal injury in which large amounts of myoglobin accumulate in the renal tubules, presenting as muscle pain, reddish-brown urine, and elevated creatine kinase levels. Our case describes a 60-year-old male who came to the emergency department with fevers, mild abdominal pain, and constitutional symptoms one day after returning to the United States from a trip to Nigeria. Initial workup demonstrated an acute kidney injury and elevated aminotransferase levels and the patient was started onatovaquone-proguanil for possible malaria given a recent diagnosis in Nigeria. Two days later, the patient was found to have rhabdomyolysis, resulting in a renal biopsy that showed myoglobin cast nephropathy. Previous literature has suggested mechanisms for the development of rhabdomyolysis in malarial infection, including inflammatory processes, direct effect of parasite accumulation, and drug-induced toxicity. Our case further implicates antimalarial therapy as a cause of rhabdomyolysis and increases awareness of myoglobin cast nephropathy as a potential complication of malaria.

## 1. Introduction

Rhabdomyolysis is a rare complication of malarial infection that results from skeletal muscle injury and the release of intracellular muscle contents into systemic circulation. Causes of rhabdomyolysis include trauma, substance abuse, infections, and medications [1]. Rhabdomyolysis may cause acute renal injury, a feature of which is the presence of myoglobin casts [2] formed from large amounts of myoglobin accumulating in the renal tubules and precipitating with Tamm–Horsfall protein [3]. In one retrospective study, 6% of the myoglobin cast nephropathy cases were due to infection [4], and malarial infection has been associated with rhabdomyolysis-induced acute renal failure in an even smaller subset of cases reported in the literature. Herein, we present a unique case of myoglobin cast nephropathy which occurred during treatment for malarial infection and in which renal biopsy played a significant role in diagnosis.

## 2. Case Presentation

A 60-year-old man with a past medical history of asthma, rheumatoid arthritis being treated with methotrexate, hypertension, and no known history of chronic renal disease presented to the emergency department with generalized weakness, intermittent fevers, nausea, vomiting, and mild abdominal pain one day after returning to the United States from a trip to Nigeria. Two days prior to leaving Nigeria, he noted the onset of intermittent fevers, was diagnosed with malarial infection, and was subsequently started on artemether-lumefantrine 80 mg/480 mg without any symptomatic improvement. The patient discontinued his artemether-lumefantrine course within the first two days of symptom onset. There was no known concurrent use of herbal supplements or plant extracts. Upon presentation to our emergency department three days after the initial symptom onset, the patient was hemodynamically stable and had an unremarkable physical exam. Complete blood counts, *Plasmodium* species antigen blood tests, *Plasmodium falciparum* antigen blood tests, light microscopy of thick and thin blood smears, respiratory infection PCR panel, and hepatitis viral antigen and antibody panel were all unremarkable. These results indicated possible but unconfirmed suppression of malarial infection by the time of testing. Serum aspartate aminotransferase (AST) and alanine aminotransferase (ALT) enzymes were elevated at 1,331 U/L and 259 U/L, respectively (AST: normal range = 10–50 U/L; ALT: normal range = 5–50 U/L). Serum creatinine was elevated at 2.57 mg/dL and blood urea nitrogen was elevated at 29 mg/dL (normal range = 8–23 mg/dL), indicating intrinsic acute kidney injury (AKI). He had an elevated protein/creatinine ratio of 74 mg/g (normal range = 0–30 mg/g). Abdominal ultrasound and computed tomography (CT) were unremarkable for abdominal pathology, but both abdominal CT and chest X-ray revealed focal interstitial thickening and ground-glass infiltrates consistent with pneumonia in the left lower lung lobe.

The patient was admitted and immediately started on atovaquone-proguanil for possible malaria, and the following day, he was initiated on azithromycin and ceftriaxone for suspected pulmonary infection and intravenous fluids for acute kidney injury. On the third day of the admission (two days after initiation of treatment and six days since symptom onset), the patient was found to have rhabdomyolysis with creatine kinase (CK) level above 19,000 U/L and worsening liver function tests, prompting aggressive hydration with an increase in 5% dextrose, 0.45% sodium chloride intravenous fluid infusion rate from 75 mL/hr to 150–175 mL/hr.

**Figure 1:**
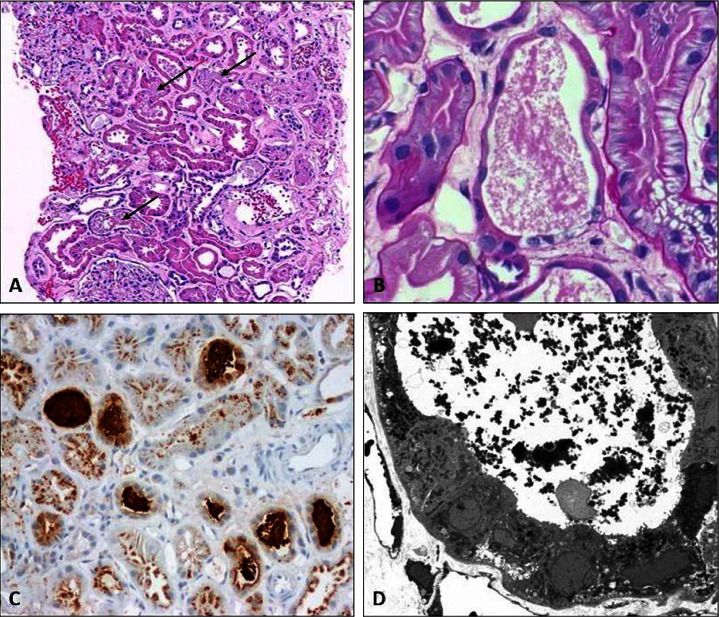
(A) Kidney biopsy showing acute tubular injury with numerous granular casts (arrows) (H&E stain, 100x). (B) PAS-stained section showing a tubule containing granular cast material (PAS, 600x). (C) Multiple myoglobin casts staining positively by immunohistochemistry (IHC-stained section, 200x). (D) Very dark acellular material within tubules corresponding to myoglobin casts (electron microscopy photomicrograph, 3000x).

In light of worsening acute kidney function and proteinuria, a percutaneous renal needle biopsy was done. The biopsy showed 20 glomeruli, one of which was obsolescent. Glomerular tufts were slightly enlarged and were without increased cellularity, segmental sclerosis, subepithelial spikes on Jones silver stain, thrombi, or necrosis. No significant interstitial inflammatory infiltrates or edema were present. Blood vessels showed mild arteriosclerosis and were without vasculitis. There was evidence of acute tubular injury consisting of tubular epithelial attenuation and flattening with marked tubular cell proliferation, and Mib-1 immunohistochemical stain showed increased proliferative activity of tubular cell nuclei. There were many tubular granular eosinophilic casts (Figures 1(A) and 1(B)) which stained positively for myoglobin on immunohistochemistry (Figure 1(C)). Immunofluorescence was weakly positive for immunoglobulin M (IgM) and complement component 3 (C3) in glomeruli (1+ segmental mesangial staining each) and negative for immunoglobulin G (IgG), immunoglobulin A (IgA), complement component 4 (C4), complement component 1a (C1q), Kappa light chain, and Lambda light chain. Electron microscopy was essentially unremarkable in glomeruli and did not show podocyte foot process effacement, electron-dense deposits, or glomerular basement membrane reduplication. There was nodular hyaline deposition in one small artery. Clumps of very dark acellular material were seen filling tubules, corresponding to the myoglobin casts seen on light microscopy (Figure 1(D)).

Over his seven-day hospital course, the patient's acute kidney injury began to resolve without the need for dialysis and he completed the standard course of atovaquone-proguanil. Prior to discharge, most laboratory values had improved. Creatinine was 3.68 mg/dL from a peak of 4.45 mg/dL, blood urea nitrogen (BUN) was 43 mg/dL from a peak of 61 mg/dL, AST was 667 U/L from a peak of 2,638 U/L, and ALT was 398 U/L from a peak of 697 U/L. CK remained >19,000 U/L. He was discharged after a nine-day hospital stay with a seven-day course of oral levofloxacin to cover for the initially suspected infectious pneumonia.

## 3. Discussion

**Figure 2:**
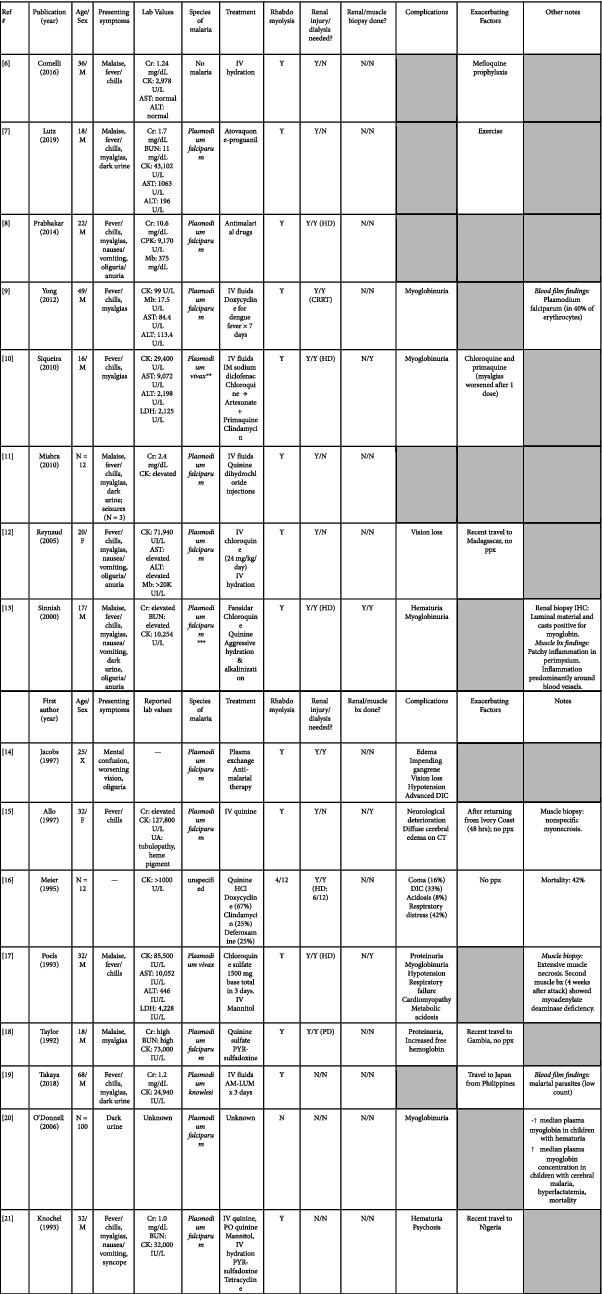
Summary of cases of rhabdomyolysis associated with malarial infection noted in previous literature. ^*∗*^Bx: biopsy, M: male, F: female, Cr: creatinine, BUN: blood urea nitrogen, CK: creatine kinase, AST: aspartate aminotransferase, ALT: alanine aminotransferase, Mb: myoglobin, Y: yes, N: no, IV: intravenous, PO: per os (oral), CRRT: continuous renal replacement therapy, HD: hemodialysis, PD: peritoneal dialysis, CT: computed tomography, DIC: disseminated intravascular coagulation, AM-LUM: artemether-lumefantrine, PYR: pyrimethamine, IHC: immunohistochemistry, ICU: intensive care unit, RBC: red blood cells, Ppx: prophylaxis, ^*∗∗*^thick blood smear negative for *Plasmodium* species on day 3 of antimalarial treatment, and ^*∗∗∗*^in 250 of 1000 erythrocytes.

The first reported case of acute rhabdomyolysis associated with malarial infection was by De Silva et al. in 1988 [5]. Several patients since then were reported to develop rhabdomyolysis in association with malarial infection, with [6–18] or without [19–21] the development of renal impairment. Infectious malarial species in this setting included *Plasmodium falciparum* [7–9, 11–15, 18, 20, 21], *Plasmodium knowlesi* [19], and *Plasmodium vivax* [10, 17].  Figure 2 summarizes the previously reported cases of rhabdomyolysis in malarial infection.

There are several possible mechanisms by which rhabdomyolysis may occur in malarial infection. A direct cause of muscle injury may be the accumulation of malarial parasites in skeletal muscle. This hypothesis is supported by the findings that increasing levels of malarial parasitemia correlated with serum myoglobin and CPK levels in a study of 58 Gambian children with malaria [22] and by a study confirming the presence of malarial parasites in skeletal muscle tissue in 36 patients with cerebral falciparum malaria [23]. The patient's elevated ALT and acute kidney injury present on admission support the hypothesis that rhabdomyolysis was a result of the malarial infection itself.

The inflammatory response in malarial infection may also cause or contribute to the development of rhabdomyolysis. Muscle biopsy in a patient with malarial infection showed increased numbers of CD4-positive T-lymphocytes, which are known to produce tumor necrosis factor alpha (TNF-a), interferon, and cytokines [13]. High-grade fever in malaria may also lead to the release of TNF-a, a known myotoxin [24]. The subsequent development of renal failure secondary to myoglobinemia and deposition of myoglobin in the kidney may be exacerbated by an inflammatory response in the kidney, as was seen on renal biopsy in one case [13]. Interestingly, in our case, no significant interstitial inflammation was seen on renal biopsy, thus showing that renal impairment may still follow rhabdomyolysis without a significant inflammatory response in the kidney itself.

Our patient was found to have rhabdomyolysis after antimalarial therapy and no detectable malarial infection on admission, raising the possibility that rhabdomyolysis could have occurred as an effect of antimalarial therapy. *Plasmodium* species antigen blood tests, *Plasmodium falciparum* antigen blood tests, and thick and thin blood smears were used to screen the patient for malarial infection on admission and were all negative for active parasitemia. Light microscopy of thick and thin blood smears is considered the gold standard for diagnosis, with thick smears able to detect a parasite load as low as 10–50 trophozoites/*µ*l, while the sensitivity of antigen testing is limited for parasite loads below 100 parasites/*µ*l [25]. Previous case reports suggest treatment with antimalarial drugs as a potential cause of rhabdomyolysis, including a case of a 36-year-old man who started antimalarial prophylaxis with mefloquine prior to a trip to Nigeria [6]. After several weeks of taking this medication, he developed malaise and fatigue, and a workup revealed elevated creatine kinase at 2,978 U/L and elevated creatinine at 1.24 mf/dL [6]. Medical management with fluid resuscitation lowered his creatine kinase levels and normalized creatinine [6]. Another antimalarial drug, chloroquine, is also hypothesized to cause muscle damage by two mechanisms. Chloroquine accumulation over time can lead to vacuolar myopathy, a condition in which autophagic vacuoles develop in muscle tissue on the ultrastructural level [26]. In addition, parasite killing by the drug can exacerbate inflammatory response, resulting in six documented cases of toxic myopathy and neuropathy after long-term administration of chloroquine over several years [17]. Our patient took artemether-lumefantrine and atovaquone-proguanil, which have not previously been implicated as a cause of rhabdomyolysis occurring in the setting of malarial infection. In addition, some antimalarial plant extracts (*Sida acuta* (PSA), *Malvaceae*, and *Enantia polycarpa*) used in conjunction with the antimalarial drugs artesunate-amodiaquine and artemether-lumefantrine have been noted to cause renal, hepatic, and neurological damage [27] though the use of these supplements was not noted in our patient.

Another explanation of rhabdomyolysis occurring following the resolution of detectable parasitemia is that myoglobin can influence renal function by precipitating in the loop of Henle [23]. Myoglobin is not detectable in the urine until serum concentrations are greater than 15,000 U/L, allowing for an insidious onset and late presentation of renal symptoms [23]. This emphasizes the importance of recognizing the possibility of rhabdomyolysis and renal impairment in patients even after the resolution of malarial infection.

Secondary factors in specific cases may also contribute to the development of rhabdomyolysis and renal injury in malaria. A case was reported of a football player who traveled to Nigeria, reported noncompliance with malarial prophylaxis, and developed rhabdomyolysis and renal failure in the setting of *Plasmodium falciparum* malarial infection. The authors of this report concluded that rhabdomyolysis and renal failure in this patient were a consequence of a combination of malarial infection with exertional effects [7]. Other secondary factors that may contribute to the development of renal failure in malarial infection are hypovolemia, excessive hemolysis, disseminated intravascular coagulopathy, and impaired microcirculation due to high levels of parasitized erythrocytes [28].

Of all the reports of rhabdomyolysis in malarial infection in the literature, only another one to our knowledge reported on findings of a renal biopsy [13]. In our case, a renal biopsy confirmed myoglobin-induced injury as the cause of acute renal failure. This is especially important when one considers the many other causes and factors that may lead to renal failure in malarial infection [29]. Renal biopsy may also rule out other causes of acute kidney injury that may be unrelated to malarial infection, including glomerulonephritis after immune complex deposition, which was also ruled out in our case given negative findings on immunofluorescence microscopy.

In conclusion, we present a rare case of myoglobin cast nephropathy in malarial infection. Our case highlights the important contribution of renal biopsy in reaching the diagnosis, possibly implicating antimalarial therapy as the cause of rhabdomyolysis, and focuses on awareness of myoglobin-induced renal failure as a complication of malaria [30].

## Data Availability

Data sharing is not applicable to this article as no datasets were generated or analyzed during this case review.

## References

[1] Zutt R., van der Kooi A. J., Linthorst G. E., Wanders R. J., de Visser M. (2014). Rhabdomyolysis: review of the literature. *Neuromuscular Disorders*.

[2] Najafian B., Fogo A. B., Lusco M. A., Alpers C. E. (2017). AJKD atlas of renal pathology: myoglobin cast nephropathy. *American Journal of Kidney Diseases*.

[3] Bosch X., Poch E., Grau J. M. (2009). Rhabdomyolysis and acute kidney injury:1982. *New England Journal of Medicine*.

[4] Liapis H., Boils C., Hennigar R., Silva F. (2016). Myoglobin casts in renal biopsies: immunohistochemistry and morphologic spectrum. *Human Pathology*.

[5] De Silva H. J., Goonetilleke A. K., Senaratna N. (1988). Skeletal muscle necrosis in severe falciparum malaria. *British Medical Journal*.

[6] Comelli I., Lippi G., Magnacavallo A., Cervellin G. (2016). Mefloquine-associated rhabdomyolysis. *The American Journal of Emergency Medicine*.

[7] Lutz R. H., Anderson McNeil J., Odo C. P. (2019). Rhabdomyolysis and malaria in a college football player. *Current Sports Medicine Reports*.

[8] Prabhakar P., Rathore S. S., Choudhury T. A., Kishan A., Gupta T., Prakash J. (2014). Rhabdomyolysis induced acute renal failure: a rare complication of falciparum malaria. *Journal of the Association of Physicians of India*.

[9] Yong K. P., Tan B. H., Low C. Y. (2012). Severe falciparum malaria with dengue coinfection complicated by rhabdomyolysis and acute kidney injury: an unusual case with myoglobinemia, myoglobinuria but normal serum creatine kinase. *Bone Marrow Concentrate Infectious Diseases*.

[10] Siqueira A. M., Alexandre M. A., Mourão M. P. (2010). Severe rhabdomyolysis caused by Plasmodium vivax malaria in the Brazilian Amazon. *The American Journal of Tropical Medicine and Hygiene*.

[11] Mishra S. K., Pati S. S., Mahanta K. C., Mohanty S. (2010). Rhabdomyolysis in falciparum malaria--a series of twelve cases (five children and seven adults). *Tropical Doctor*.

[12] Reynaud F., Mallet L., Lyon A., Rodolfo J. M. (2005). Rhabdomyolysis and acute renal failure in Plasmodium falciparum malaria. *Nephrology Dialysis Transplantation*.

[13] Sinniah R., Lye W. (2000). Acute renal failure from myoglobinuria secondary to myositis from severe falciparum malaria. *American Journal of Nephrology*.

[14] Jacobs P., Wood L., Barendse G., Maresky A. (1997). Reversal of life-threatening vascular occlusion by apheresis in fulminating Plasmodium falciparum malaria. *Hematology*.

[15] Allo J. C., Vincent F., Barboteu M., Schlemmer B. (1997). Falciparum malaria: an infectious cause of rhabdomyolysis and acute renal failure. *Nephrology Dialysis Transplantation*.

[16] Meier S., Krause M., Lüthy R., Baumann P. C., Markwalder K. (1995). Intensivmedizinische Aspekte bei schwerer Malaria tropica: klinik, Therapie und prognostische Faktoren [Intensive care aspects in severe tropical malaria: clinical aspects, therapy and prognostic factors]. *Schweizerische Medizinische Wochenschrift*.

[17] Poels P. J., Dolmans W. M., Gabreëls F. J. (1993). Rhabdomyolysis associated with malaria tertiana in a patient with myoadenylate deaminase deficiency. *Tropical and Geographical Medicine*.

[18] Taylor W. R., Prosser D. I. (1992). Acute renal failure, acute rhabdomyolysis and falciparum malaria. *Transactions of the Royal Society of Tropical Medicine and Hygiene*.

[19] Takaya S., Kano S., Kutsuna S., Suzuki T., Komaki-Yasuda K., Ohmagari N. (2018). Case report: *Plasmodium knowlesi* infection with rhabdomyolysis in a Japanese traveler to palawan, the Philippines. *The American Journal of Tropical Medicine and Hygiene*.

[20] O’Donnell A., Weatherall D. J., Taylor A. M., Reeder J. C., Allen S. J. (2006). Muscle cell injury, haemolysis and dark urine in children with falciparum malaria in Papua New Guinea. *Transactions of the Royal Society of Tropical Medicine and Hygiene*.

[21] Knochel J. P., Moore G. E. (1993). Rhabdomyolysis in malaria. *New England Journal of Medicine*.

[22] Miller K. D., White N. J., Lorr J. A., Roberts J. M., Greenwood B. M. (1989). Biochemical evidence of muscle injury in African children with severe malaria. *Journal of Infectious Diseases*.

[23] Davis T. M., Pongponratan E., Supanaranond W. (1999). Skeletal muscle involvement in falciparum malaria: biochemical and ultrastructural study. *Clinical Infectious Diseases*.

[24] Flores E. A., Bistrian B. R., Pomposelli J. J., Dinarello C. A., Blackburn G. L., Istfan N. W. (1989). Infusion of tumor necrosis factor/cachectin promotes muscle catabolism in the rat. A synergistic effect with interleukin 1. *Journal of Clinical Investigation*.

[25] Wangai L. N., Karau M. G., Njiruh P. N. (2011). Sensitivity of microscopy compared to molecular diagnosis of p. Falciparum: implications on malaria treatment in epidemic areas in Kenya. *African Journal of Infectious Diseases*.

[26] Casado E., Gratacós J., Tolosa C. (2006). Antimalarial myopathy: an underdiagnosed complication? Prospective longitudinal study of 119 patients. *Annals of the Rheumatic Diseases*.

[27] Daubrey-Potey T., Adjogoua V., Kamagaté M., Aoussi S., Dosso M. (2021). Artemisinin-based combination therapy synergized with medicinal plants to induce musculotoxic effects. *Evidence-based Complementary and Alternative Medicine*.

[28] Eiam-Ong S. (2003). Malarial nephropathy. *Seminars in Nephrology*.

[29] Brown D. D., Solomon S., Lerner D., Del Rio M. (2020). Malaria and acute kidney injury. *Pediatric Nephrology*.

[30] El-Zaatari Z. M., Sutaria N. B., Truong L., Barrios R., Gaber L. W. Myoglobin cast nephropathy diagnosed on renal biopsy in a patient treated for malarial infection (poster No. 120).

